# Falsely decreased triglyceride concentration in a patient with acute pancreatitis due to insufficient configuration of alarm rules

**DOI:** 10.11613/BM.2026.011002

**Published:** 2026-02-15

**Authors:** Yin Liu, Qianhui Liu, Bin Feng, Wei Gan

**Affiliations:** 1 Department of Laboratory Medicine, Clinical Laboratory Medicine Research Center, West China Hospital, Sichuan University; Sichuan Clinical Research Center for Laboratory Medicine, Chengdu, Sichuan Province, China; 2 Sichuan University, West China Clinical Medical College, Chengdu, Sichuan Province, China

**Keywords:** acute pancreatitis, analytical techniques and equipment, analyzer alarms, case report, hypertriglyceridemia

## Abstract

Acute pancreatitis is a potentially life-threatening complication of severe hypertriglyceridemia. The accurate measurement of triglyceride concentration is essential for diagnosis and therapeutic monitoring. This article presents a case of acute hypertriglyceridemia pancreatitis in which the patient’s triglyceride concentration appeared to drop rapidly from 128.04 mmol/L to 1.53 mmol/L within 12 hours of admission. Subsequent retesting revealed an actual triglyceride value of 88.92 mmol/L. This case underscores a critical and underreported preanalytical challenge: the failure of the analyzer to trigger “> Kin” warnings despite clear abnormalities in the reaction kinetics curve, leading to clinically significant underestimation. To mitigate such errors, we propose a novel strategy that integrates automated lipemic index-based predilution protocols with enhanced alarm configurations, including the introduction of a “>Abs” alert, adjustment of prozone detection parameters, and the implementation of correlation checks between lipemic indices and triglyceride values within the laboratory information system. These practical interventions, which can be adopted in clinical laboratories, represent a proactive approach to prevent erroneous reporting and enhance diagnostic reliability. This report highlights the necessity for increased vigilance among laboratory professionals when discordance occurs between high lipemic indices and unexpectedly low triglyceride results, suggesting possible kinetic anomalies.

## Graphical abstract

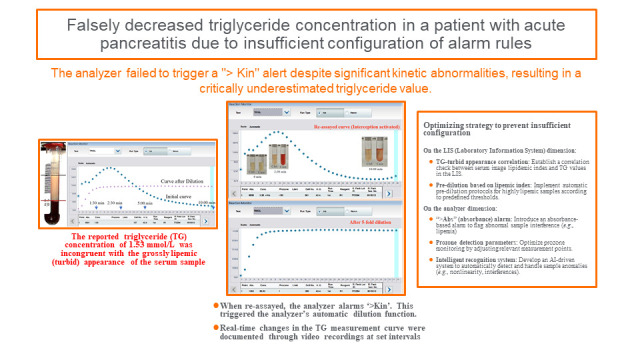

## Highlights

• Kinetic instability in high-triglyceride (TG) samples: rapid chromogen formation followed by bleaching due to oxygen depletion

• Inadequate analyzer alarm rules failed to flag extreme TG reaction curves

• Laboratory professionals should recognize falsely low TG from abnormal curves

• Laboratory information system-based correlation monitoring: lipemic index and TG value verification

## Introduction

Hypertriglyceridemia (HTG), defined as a fasting triglyceride (TG) concentration exceeding 1.7 mmol/L, represents the third most common cause of acute pancreatitis after gallstones and excessive alcohol consumption (*1*). It is further classified as severe when TG concentrations exceed 10 mmol/L and very severe when TG concentrations reach or exceed 20 mmol/L (*2*). Recent studies indicate that hypertriglyceridemia-induced pancreatitis (HTGP) may account for up to 22% of all acute pancreatitis cases, particularly among patients with markedly elevated baseline triglycerides (*3*). Notably, HTGP is associated with higher mortality compared to other etiologies of pancreatitis, and elevated triglyceride concentrations after discharge are strongly correlated with the risk of recurrence (*4*, *5*). This underscores the critical importance of early recognition and aggressive triglyceride-lowering therapy, with current guidelines recommending maintaining serum triglycerides below 5.65 mmol/L during acute management and under 2.27 mmol/L for long-term prevention of recurrence (*6*, *7*).

Accurate measurement of triglyceride concentrations is therefore essential not only for diagnosis but also for guiding therapeutic interventions such as plasmapheresis, insulin infusion, and other TG-lowering treatments aimed at interrupting the vicious cycle of inflammation and hypertriglyceridemia (*8*). Over recent decades, the clinical measurement of triglycerides has benefited from improved analytical efficiency, quality control, and standardization through the widespread adoption of automated biochemical analyzers (*9*). These systems incorporate technical alarm functions to detect preanalytical and analytical errors, including sample clots, reagent insufficiency, instrument failure, and reaction abnormalities, thereby reducing the reporting of erroneous results, particularly those caused by interference (*10*-*12*). However, despite these advancements, rare but clinically significant errors persist, such as falsely low triglyceride results due to inadequately configured analytical monitoring systems, as observed with the Roche Cobas c702 analyzer (Roche Diagnostics GmbH, Mannheim, Germany).

## Laboratory analyses

**Figure 1:**
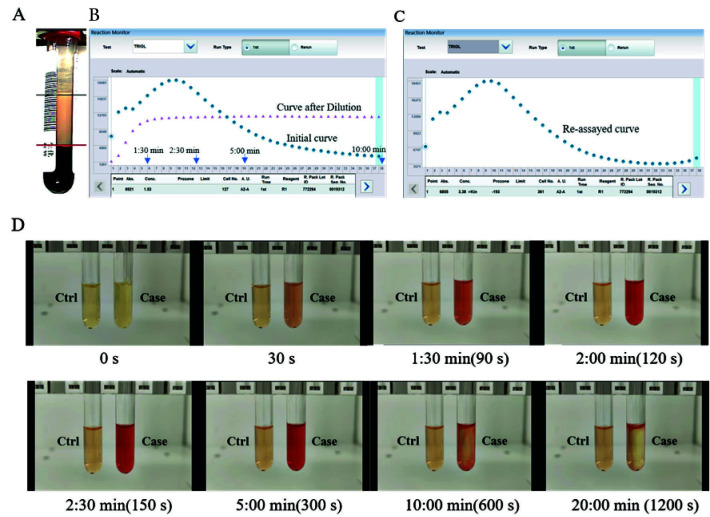
Patient plasma sample and the kinetics curve. (A) Plasma sample visualization; (B) The kinetics curve of falsely result and post-dilution; (C) The kinetics curve of re-measurement; (D) Screenshots of the video in triglycerides measurement of hyperlipidemia specimens at different time points; Control: 2.20 mmol/L, Case: 88.92 mmol/L.

An older male patient presented to the emergency department of West China Hospital with left abdominal distension, pain and discomfort from a high-fat diet four hours prior, accompanied by vomiting yellow fluid 3 times. On admission to our hospital, blood samples were collected using serum collection tubes (red cap) (Becton, Dickinson & Company (BD), Franklin Lakes, USA). After clotting, the serum was separated by centrifugation at 3500*xg* for 10 minutes at room temperature. Serum lipids, electrolytes and pancreatitis markers were measured via a Roche Cobas c702 analyzer and matching reagents. The results were as follows: triglyceride (TG): 128.04 mmol/L; total cholesterol (TC): 29.42 mmol/L; amylase: 133 U/L; lipase: 382 U/L and pancreatic amylase: 116 U/L (Table 1). Together with an abdominal imaging report and clinical symptoms, HTGP was diagnosed, and intravenous insulin was used to lower the TG concentration along with heparin. During the following 12 hours, the pancreatic enzyme activities continued to increase (serum lipase increased to 681 U/L, pancreatic amylase to 198 U/L), and the TC decreased to 22.02 mmol/L, whereas TG sharply decreased to 1.53 mmol/L (Table 1). This TG result is perplexing because it is discordant with both the serum’s grossly turbid appearance (Figure 1A) and the objective artificial intelligence (AI)-based lipemia index analysis. The pre-analytical instrument (which automatically analyzes serum images, generates a Hemolysis, Icterus, Lipemia (HIL) index, and transmits it to the Laboratory Information System - LIS) graded the sample as ‘lipemia ++++,’ a finding that typically confirms severe lipemia.

**Table 1:** Patient laboratory results

**Parameter, unit**	**At admission**	**After 12 hours**
Triglycerides, mmol/L (reference interval: 0.29-1.7)	128.04	1.53 (88.92 after dilution)
Cholesterol total, mmol/L (reference interval: 2.8-5.17)	29.42	22.02
High-density lipoprotein cholesterol, mmol/L (reference interval: ≥ 1)	0.12	0.17
Low-density lipoprotein cholesterol, mmol/L (reference interval: < 3.4)	0.72	0.52
Amylase, U/L (reference interval: 35-135)	133	216
Lipase, U/L (reference interval: 13-60)	382	681
Pancreatic amylase, U/L (reference interval: 13-53)	116	198

## Further investigation

To determine the cause, we checked the kinetic curve of the TG in analyzer and found that it was obviously abnormal, but the analyzer still gave a result of 1.53 mmol/L without triggering any alarms (Figure 1B). The technician identified this inconsistency. The sample was subsequently reassayed, and the analyzer displayed a ‘> Kin’ alarm, indicating kinetic instability (Figure 1C). This triggered the analyzer’s automatic dilution function. After automatic 5-fold dilution by the analyzer, the actual value of the serum triglyceride concentration was determined to be 88.92 mmol/L, significantly higher than the initial reported value of 1.53 mmol/L (Table 1).

## What happened?

To capture the dynamic measurement curve of the TG for this sample, a video was recorded (see in Supplementary material). After adding the reagent, the red products rapidly generated and reached peak intensity at 150 seconds (corresponding to approximately the 10th point on the dynamic curve). Subsequently, the color became uneven and gradually faded. However, in the normal control, the solution reached peak intensity at 90 s (approximately the 6th point) and remained stable thereafter. When the end-point (10 minutes) was reached, the red-colored solution appeared uneven, with numerous red particles floating on the surface. When the reaction time was extended to 20 minutes, the red color in the solution disappeared, leaving only a significant accumulation of color at the surface (Figure 1D). The surface, being in contact with the air, maintains a continuous supply of oxygen, allowing the reaction to proceed. As a result, an accumulation of the red-colored product (benzquinamide) is observed there. However, O_2_ in the solution is scarce, and the concentration of benzquinamide compounds gradually decreases. The continuous decrease in the reaction kinetics curve for the case sample is consistent with the disappearance of the red color in the video. Therefore, it is important to identify abnormal reaction by kinetic monitoring to intercept erroneous results. Why did the analyzer fail to trigger a “> Kin” alarm during the initial test?

The kinetic monitoring of triglycerides on Roche systems employs a “>Kin” alarm based on rate changes in absorbance at four prozone measuring points (pmp), a process referred to as the Prozone Check (PC). The algorithm analyzes absorbance readings at pmp2, pmp4, pmp10, and pmp14 through three sequential steps (Table 2):

**Table 2:** Prozone check result calculation display

**First Step** **(Calculation absorbance (Primary - Secondary))**			
**Prozone measuring** **points**	**Initial curve** **(Interception not** **activated)**	**Reassayed curve** **(Interception activated)**	**Curve after dilution**
pmp_2_	12335	12137	2594
pmp_4_	13024	13329	8082
pmp_10_	19480	19450	11098
pmp_14_	15021	14849	11217
**Second Step** **(Calculation absorbance between pmp4 and pmp2 or pmp14 and pmp10)** **if |Apmp4 - Apmp2| < 1000, the reaction rate prozone limit check is not performed**			
Apmp_14_ - Apmp_10_	- 4459	- 4601	119
Apmp_4_ - Apmp_2_	689 (|Apmp4 - Apmp2| < 1000 limit check not performed)	1192	5488
**Third Step** **(Prozone limit check)** **prozone limits from -3 to 100, out of this range triggering “>Kin” alarm**			
V2((Apmp_14_-Apmp_10_)/4)		- 1150.25	29.75
V1((Apmp_4_-Apmp_2_)/2)	Not performed	596	2744
PC = V2/V1*100		- 192.99 (out of the prozone limits(- 3 to 100))	1.08 (In the prozone limits)

Step 1: The absorbance value at each pmp is calculated by subtracting the secondary wavelength reading from the primary wavelength.

Step 2: Absorbance differences are computed. The absolute difference between pmp4 and pmp2 (|Apmp4 - Apmp2|) is evaluated to determine whether the reaction exhibits a sharp change due to high triglyceride (TG) concentration.

Step 3: Prozone limit check. This step is activated only if the difference from Step 2 reaches or exceeds 1000. The algorithm then calculates the ratio of the late-phase reaction rate (between pmp14 and pmp10, denoted as V_2_) to the early-phase rate (between pmp4 and pmp2, denoted as V_1_), multiplies the result by 100, and checks whether it falls within the range of - 3 to 100. Values within this range are considered acceptable and do not trigger an alarm, while values outside this range confirm a significant kinetic abnormality, activate the “>Kin” alarm, and invalidate the result.

In the case presented here (Table 2), the initial reaction measurement was not flagged by the “>Kin” alarm. The absorbance values at the critical points were Apmp2 = 12,335 and pmp4 = 13,024. The absolute difference |13,024 – 12,335| = 689 fell below the threshold of 1000, indicating that the reaction did not show a sufficiently sharp change to suggest a high TG concentration. As a result, the condition for initiating Step 3 was not met, and the prozone limit check was bypassed. The triglyceride concentration was subsequently calculated using the endpoint method, yielding an erroneous result of 1.53 mmol/L. Notably, although the raw reaction data showed a visually conspicuous deviation in the kinetic curve, which should have triggered the “>Kin” alarm, the analyzer’s internal monitoring algorithm failed to recognize this anomaly. Consequently, no “>Kin” flag was generated, and the analyzer transmitted the erroneous result without any critical alert to the LIS. In the absence of an error flag from the analyzer, the LIS accepted the result without further interrogation according to its standard operating procedure. The core issue, therefore, lies in the failure of the analyzer’s software algorithm to reliably detect abnormal reaction kinetics under these specific conditions, resulting in a false-negative error reporting.

## Discussion

Triglycerides (TG) were quantified using a one-step enzymatic colorimetric assay (Roche Diagnostics). The method is based on a coupled enzymatic cascade leading to the formation of a chromogenic product. Specifically, serum triglycerides are enzymatically hydrolyzed to release free glycerol. The glycerol is subsequently phosphorylated by adenosine triphosphate (ATP) in a reaction catalyzed by glycerol kinase, yielding glycerol-3-phosphate. This intermediate is then oxidized by glycerol-3-phosphate oxidase, generating hydrogen peroxide (H_2_O_2_) as a byproduct. Finally, the hydrogen peroxide drives a Trinder-type endpoint reaction in the presence of peroxidase, where it oxidatively couples 4-aminophenazone and 4-chlorophenol to form a red quinoneimine dye. The intensity of this chromogen is measured spectrophotometrically at a primary wavelength of 505 nm, with a secondary reference wavelength of 700 nm to correct for background interference. Oxygen and water are essential co-substrates in this reaction sequence (*13*). A typical reaction curve is illustrated in Figure 1B (post-dilution curve). However, in samples with elevated triglyceride concentrations, substrates, particularly oxygen, are rapidly depleted. Under anaerobic conditions, the enzymatic pathway may be altered, and preformed chromogen can undergo reduction (a phenomenon known as “bleaching”) by serving as an alternative electron acceptor for glycerol-3-phosphate oxidase. This leads to underestimation of the true triglyceride concentration (*14*). Since all commercially available triglyceride assays rely on this same chemical principle, chromogen bleaching represents a universal challenge in clinical chemistry, particularly in samples with high triglyceride concentrations. Although different analytical platforms employ varying algorithms to detect abnormal reaction kinetics, those using monitoring strategies and logic similar to the Roche system, which depends primarily on prozone checking, remain prone to erroneously reporting falsely low results in hypertriglyceridemic samples. This is because the activation of alerts under such monitoring logic exhibits inherent randomness and lacks consistent reliability. As shown in Figure 1B (initial curve), absorbance changes at early time points (*e.g*., points 2 and 3) are discordant. The value at point 2 rises, resulting in an absorbance difference between pmp4 and pmp2 of less than 1000. Consequently, the prozone limit check is not triggered (Table 2). These findings highlight that reliance solely on the “>Kin” alarm is insufficient for reliable kinetic monitoring of high‐triglyceride samples on the Cobas c702 system.

The limitations described above are not merely theoretical. Failure to trigger triglyceride prozone checks in severely lipemic samples appears to be an underrecognized issue on the Cobas c702 platform. In addition to the case presented here, three previous studies have also documented falsely low triglyceride concentrations in lipemic plasma (*15*-*17*). While these studies discussed the causes of such inaccuracies and emphasized the clinical importance of recognizing pseudolow triglycerides in hypertriglyceridemia, none identified potential technical flaws in the front‐area inspection of the kinetic curve. Notably, we strongly suspect that at least one of these previously reported cases may have been due to a prozone check failure, given that the same analytical system (Cobas c702) was used (*17*). To the best of our knowledge, this is the first report to attribute pseudolow triglyceride results specifically to an alarm system loophole accompanied by significant kinetic curve abnormalities.

To mitigate errors resulting from prozone check failures in intercepting high-concentration triglyceride samples on analyzers such as the Cobas c702 or other systems with similar logic, the implementation of enhanced detection and preprocessing strategies is recommended. Based on current technological capabilities, the following multidimensional approaches should be considered:

At the LIS level:

1. Establish an automated correlation check between the serum lipemic index and TG values. Significant discrepancies should trigger alerts during result verification, prompting technologists to visually inspect sample appearance, evaluate reaction kinetics, and/or perform repeat testing.
2. Implement rule-based automatic pre-dilution protocols for samples exhibiting lipemic indices above predefined thresholds (*e.g.,* ≥ +++), utilizing integrated HIL index data obtained from automated biochemical analyzers or image-based AI recognition systems.

At the analyzer level:

1. Introduce an absorbance-based alarm mechanism to identify abnormal reaction kinetics. For instance, using the Roche Cobas c702 analyzer, setting an absorbance limit (> 12,000) at the 6th measurement point can effectively flag severely lipemic samples, since their peak absorbance typically occurs later (*e.g*., at point 10) than in normal samples.
2. Optimize prozone detection parameters by expanding the number of monitoring points and refining criteria, such as setting a delta threshold between endpoint and maximum absorbance.
3. Develop an intelligent kinetic curve recognition system using adaptive machine learning to identify nonlinearity and interference patterns. Unlike static rule-based algorithms, adaptive AI enhances continuous pattern learning and provides greater flexibility in anomaly detection, as demonstrated in reaction monitoring and pattern recognition (*18*-*20*).

To enhance the detection and preprocessing of severely lipemic samples, our laboratory has implemented an image-based AI algorithm for serum index recognition over the past five years; however, it was only following this incident that a definitive protocol establishing a correlation between the lipemic index and triglyceride values was formally introduced. This integration has proven to be a practical and effective safeguard against the erroneous reporting of artificially low lipid concentrations in grossly lipemic specimens. Nevertheless, an automatic pre-dilution step triggered directly by lipemia index has not yet been deployed, as its implementation requires extensive validation of inter-instrument connectivity, clearly defined threshold criteria, and confirmation of optimal dilution factors to ensure analytical accuracy. Addressing these aspects remains a critical direction for future methodological refinement.

## What YOU can do in your laboratory to prevent such errors

To mitigate such errors within the laboratory setting, the following measures are recommended:

1. Universal HIL index assessment: Implement systematic measurement of HIL indices on all samples using automated biochemical analyzers or image-based AI assessment tools.
2. Automated pre-dilution for lipemic samples: Establish rule-based automatic pre-dilution procedures for samples exceeding predefined lipemic index thresholds (*e.g*., ≥ +++), to minimize analytical interference prior to testing.
3. LIS-based correlation monitoring: Incorporate a real-time correlation check between the lipemic index and triglyceride values within the LIS. Significant discrepancies should automatically trigger an alert during the validation phase, prompting laboratory staff to inspect reaction kinetics curves on the analyzer to verify result reliability and identify potential procedural or instrumental deviations.

In summary, improperly configured alert rules can fail to identify severely lipemic samples, resulting in spuriously low triglyceride values. Laboratorians should acknowledge the inherent limitations of kinetic algorithms in handling such samples and develop strengthened protocols to reduce the risk of inaccurate triglyceride reporting.

## Data Availability

No data was generated during this study, so data sharing statement is not applicable to this article.
